# Food Systems’ Transformation to Address Malnutrition in Selected Countries—Panel-Data Analysis on Undernourishment and Obesity

**DOI:** 10.3390/foods11091323

**Published:** 2022-04-30

**Authors:** Eihab Fathelrahman, Safdar Muhammad, Afra Abdalla, Habiba I. Ali, Ayesha Al Dhaheri

**Affiliations:** 1 Department of Integrative Agriculture, College of Agriculture and Veterinary Medicine, United Arab Emirates University (UAEU), Al Ain 15551, United Arab Emirates; smuhammad@uaeu.ac.ae (S.M.); afra.abdalla@uaeu.ac.ae (A.A.); 2 Department of Nutrition and Health, College of Medicine and Health Sciences, United Arab Emirates University (UAEU), Al Ain 15551, United Arab Emirates; habali@uaeu.ac.ae (H.I.A.); ayesha_aldhaheri@uaeu.ac.ae (A.A.D.)

**Keywords:** food system, transformation, panel data, fixed and random effect model, undernourishment, obesity

## Abstract

Global awareness of the 2030 Sustainable Development Goals (SDGs) has heightened the importance and role of food systems’ transformation in accelerating countries’ progress to achieve such goals in a timely manner. The agricultural and food systems’ transformation goal is to build resilience to vulnerabilities, shocks, and diseases such as undernourishment and obesity. The objective of this study is to specify the agricultural and food systems’ factors that contribute to reducing the impacts of undernourishment and obesity in selected populous and high-income countries. This study used panel data from 2000 to 2020, applying fixed and random-effect econometrics models to conduct the analyses. Results indicated that the per capita Gross Domestic Product (GDP), level of urbanization, and flood losses at food retail from a food systems perspective were the most influential factors in the prevalence of undernourishment. Meanwhile, the supply of fatty food was the significant nutrition variable affecting undernourishment. The cereal import dependency, per capita GDP, percentage of food imports from the value of food export, and value of domestic food production factors were the influential food system variables affecting the prevalence of obesity. Cereal import dependency and dietary energy supply are the significant factors affecting the prevalence of obesity. This study recommended a set of policy actions to address malnutrition, including sustainable and balanced food and nutrition systems’ transformation, food trade openness, and efforts to reduce adversary impacts of urbanization.

## 1. Introduction

The Global Panel on Agriculture and Food Systems for Nutrition showed that poor diets are the major contributor to the global burden of disease, accounting for 20% of premature disease-caused mortality worldwide. Approximately 3 billion people cannot afford a healthy diet, and more than 3 billion people suffer from one or more types of malnutrition due to poor diets. Poor nutrition can lead to reduced earning potential and increased costs for healthcare; it locks individuals and families into inter-generational cycles of poverty, deprivation, inequality, and disadvantage around the world (Foresight 2, 2020) [1].

Obesity and overweight are severe public health issues with rapidly increasing rates in developed and developing countries. According to the World Health Organization (WHO, 2022), 1.9 billion adults (over the age of 18) were overweight in 2014, and 600 million were obese. In addition, 42 million children under five were overweight or obese in 2013 [2]. José Graziano da Silva, the Director-General of the United Nations Food and Agriculture Organization (FAO, 2022), indicated: “Today, over half of the world’s population is affected by malnutrition, be it hunger, micronutrient deficiencies, or excessive consumption.” [3].

**Figure 1:**
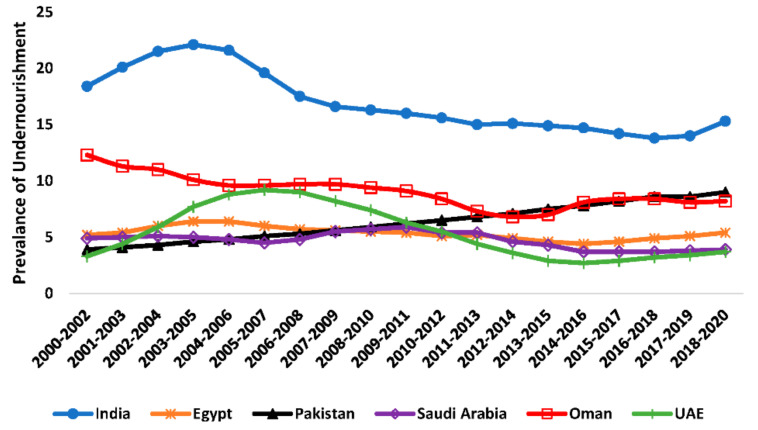
Prevalence of undernourishment in six countries (India, Pakistan, Egypt, Saudi Arabia, and the United Arab Emirates) [4].

In populous countries, such as India, Pakistan, and Egypt, the prevalence of undernourishment was reported by FAO to be 15.3% (209 million people), 9% (28 million people), and 5.4% (5 million people) of the population on average for the period of 2018–2020, respectively. The prevalence of undernourishment in oil-rich countries such as Oman, Saudi Arabia, and the United Arab Emirates (UAE) was reported to be 8.2% (0.4 million people), 3.9% (1.3 million people), and 3.7% (0.4 million people), respectively. However, it is worth mentioning that India, Saudi Arabia, Oman, and UAE’s undernourishment has declined over the last two decades. Egypt has not shown progress toward reducing undernourishment. Meanwhile, in Pakistan, the prevalence of undernourishment increased from 3.3% in 2001 to 9% of the population in 2021. The total population of undernourished individuals in the six countries selected in this study (India, Pakistan, Egypt, Saudi Arabia, Oman, and the United Arab Emirates) was approximately 235 million people in 2020—Figure 1 [4].

**Figure 2:**
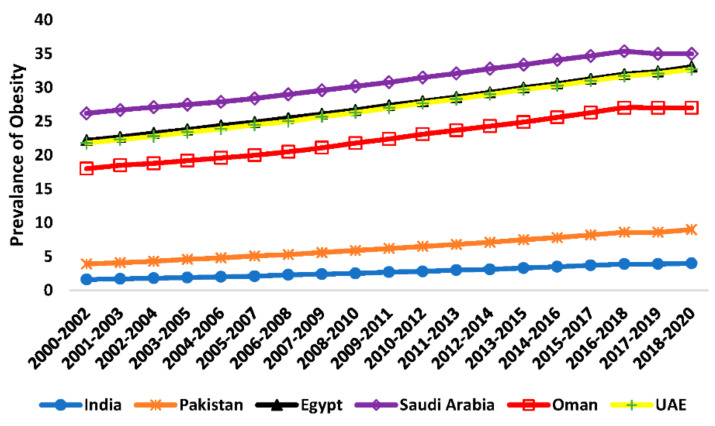
Prevalence of obesity in the six countries (India, Pakistan, Egypt, Saudi Arabia, and United Arab Emirates) [4].

The Food and Agriculture Organization (FAO) reported that obesity, measured by the prevalence of obesity for the population 18 years and older, is on the rise worldwide. The highest prevalence of obesity among this study’s selected countries was found in Saudi Arabia with 35% of the country’s population (8.1 million people), followed by Egypt at 33% (18.4 million people), the United Arab Emirates at 32% (2.5 million people), and Oman with 27% (0.9 million people), on average, for the period of 2018–2020. Meanwhile, obesity prevalence was found to be 9% (10.2 million people) in Pakistani and 4% (18.4 million people) in India. Overall, obesity is rising in all selected countries, and the obese population reached approximately 60 million people in this study’s selected countries combined—Figure 2 [4].

### 1.1. Non-Communicable Diseases (Undernourishment and Obesity) Current Situation in the Selected Countries

The Sustainable Development Goals (SDGs) reports in 2021 on the progress of Non-Communicable Diseases (NCDs) by 2030 for the selected countries in this study showed that Saudi Arabia had shown limited progress toward achieving the diet-related reduction targets for Non-Communicable Diseases (NCDs). The country has shown no progress towards achieving the target for obesity, with an estimated 45.5% of adult (aged 18 years and over) women and 34.3% of adult men living with obesity. Saudi Arabia’s obesity prevalence is higher than the regional average of 10.3% for women and 7.5% for men. Egypt has shown limited progress towards achieving the diet-related NCD targets and no progress towards achieving the target for obesity, with an estimated 44.7% of adult (aged 18 years and over) women and 25.9% of adult men living with obesity. Egypt’s obesity prevalence is higher than the regional average of 20.7% for women and 9.2% for men. Pakistan has shown limited progress towards achieving the NCD targets and no progress towards achieving the target for reducing obesity, with an estimated 13.4% of adult (aged 18 years and over) women and 7.5% of adult men living with obesity. Pakistan’s obesity prevalence is higher than the regional average of 10.3% for women but is equal to the regional average of 7.5% for men. In Oman, limited progress is seen towards achieving the diet-related NCD targets and no progress is seen towards achieving the target for obesity, with an estimated 37.1% of adult (aged 18 years and over) women and 26.4% of adult men living with obesity. Oman’s obesity prevalence is higher than the regional average of 10.3% for women and 7.5% for men. In addition, UAE had shown limited progress towards achieving the diet-related NCD targets and no progress towards achieving the target for obesity, with an estimated 44.2% of adult (aged 18 years and over) women and 30.9% of adult men living with obesity. UAE’s obesity prevalence is higher than the regional average of 10.3% for women and 7.5% for men. Finally, India has shown limited progress towards achieving the diet-related NCD targets and no progress towards achieving the target for obesity, with an estimated 6.2% of adult (aged 18 years and over) women and 3.5% of adult men living with obesity. India’s obesity prevalence is lower than the regional average of 10.3% for women and 7.5% for men [4,5]. Overall, such slow progress toward addressing the 2030 SDGs goals and higher rates of undernourishment and obesity compared to global averages are apparent in India, Pakistan, and Egypt, being populous countries, as well as in oil-rich countries compared to the Middle East and North Africa (MENA) regional averages [5].

### 1.2. Lack of Transformation in the Food System to Address Malnutrition

The region has structural challenges that make feeding a growing population particularly difficult. The first is climate change. An increase in the frequency of extreme weather and higher temperatures is affecting local agriculture. Half of the people of MENA already live under conditions of water stress, and with the population expected to grow to 700 million in 2050, per capita water availability will be halved. India and Pakistan face similar challenges in that the food system fails to support people’s health. The food provides calories but insufficient nutrition. As a result, people suffer from the double burden of both undernourishment and obesity.

**Figure 3:**
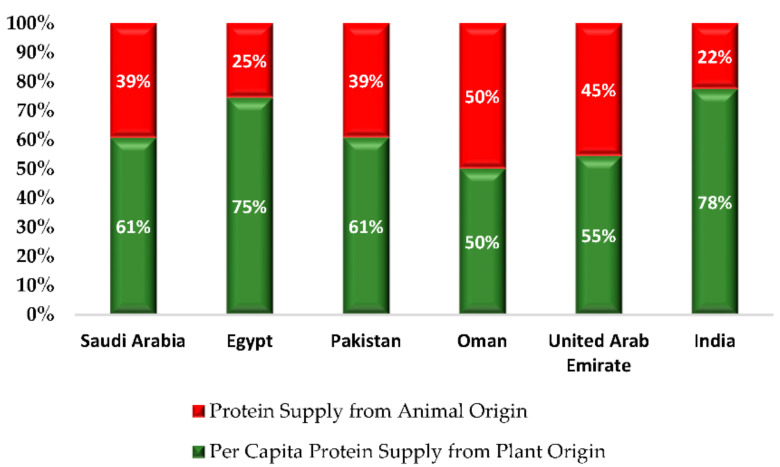
Share of plant versus animal protein source on average per capita diet.

Figure 3 shows that in India and Egypt, sources of protein in the per capita diet are mostly (over 75%) from plant origin sources, being approximately 60% in both Pakistan and Saudi Arabia, causing the reliance on protein from animal sources to be about 40%. However, such a percentage is lesser in the United Arab Emirates and Oman due to a higher reliance on animal protein sources, at 45% and 50%, respectively. Such differences between countries are indicative of differences in the populations’ diets.

The six countries selected in this study represent a unique case in connection to malnutrition challenges and food consumption patterns. However, there is the potential for strong food bilateral trade openness between them, which may contribute to a reduction in the severity of malnutrition challenges in each country. Table 1 shows the six countries selected in this study regarding bilateral food trade, which indicates that there are very strong food trade ties between India, Pakistan, and Egypt on the one hand as major exporters of food, and Saudi Arabia, UAE, and Oman as net food-importing countries on the other hand. India is the largest food exporter to the Gulf Cooperation Council (GCC) countries as exports reached approximately 5.2 billion dollars in 2020, followed by Pakistan with 1.1 billion dollars and Egypt with approximately 1.1 billion dollars’ worth of imported food by the three GCC countries (Saudi Arabia, Oman, and UAE) in 2020 [4].

In 2018, Masters et al., used a Preston Curve approach to test changes over time in agriculture, nutrition, and food policy, comparing national averages in Africa and elsewhere at each level of national income per capita from the 1990s to the 2010s. The authors showed that African countries have faster rural population growth, a larger share of workers in agriculture, and lower agricultural labor productivity than countries elsewhere. The paper presented evidence of structural shifts toward less child stunting everywhere and towards more adult obesity in high-income countries. The overall pattern of African governments’ food policies and government expenditure has not shifted. They continue price interventions and low investment levels characteristic of low-income countries worldwide [6]. Webb and Block (2012) suggested that agricultural support is the milestone for structural economic transformation and poverty reduction; however, this transformation is linked to a reduction in child undernutrition but may promote a surprisingly rapid increase in obesity [7]. Popkin (2014) tested the implications of rapid dynamic shifts in consumer purchases for food in agriculture and found that this retail sector growth will change food security, and this issue needs more research and policies [8]. Ecker (2018) studied Ghana’s agricultural transformation, food, and nutrition. The author indicated that the transformation of agriculture appears to have played an essential role in this context. However, the linkages between the agricultural transformation and food and nutrition security at the household level are not well understood. This article examines the linkage between farm production diversity and household dietary diversity in rural Ghana and how that linkage changed between 2005–2006 and 2012–2013. The empirical analysis employs a regression model that controls the region- and time-fixed effects. The estimation results suggest that farm production diversification and household income growth are related to dietary diversity [9]. Kennedy et al., (2020) presented a Food and Nutrition Security program that is strongly associated with an increased impact, resilience, sustainability, and transformation, a partnership between the European Union and Food and Agriculture Organization (FAO). The program aims to assess progress in improving food security and nutrition in 24 countries. This paper presents the results of quantitative analyses, literature reviews, country reports, and the May 2019 consensus workshop as the basis for identifying issues that must be addressed by the program in these countries going forward. Seven thematic areas were emphasized as essential for meeting the targets of Sustainability Development Goal 2 (SDG2–zero hunger). These factors include reinventing agriculture, unleashing the private sector, gender equity, decentralization of programs, multi-sector concepts within a sector approach, prioritization, data, and the political process and governance. The authors conclude that major food systems’ transformation offers new directions to fast forward progress toward SDG 2 [10]. Brourwer et al., (2021) reviewed current knowledge on drivers of consumer choices at the individual and food environment levels with special emphasis on low- and middle-income countries, discussed the converging and conflicting objectives that exist among multiple food-system actors, and argued that failure to strengthen synergies and resolve trade-offs might lead to missed opportunities and benefits of food systems’ transformation. The authors suggested that complex food systems’ transformations in evolving multiple actors, activities, and outcomes call for strong food system governance and political will to provide direction and incentives to the relevant food system agents. By adopting reverse thinking and starting from a dietary perspective to food systems’ transformation, the proposed paradigm shifts should be of relevance for the implementation [11].

Gillespie and van den Bold (2017) showed that malnutrition is a global challenge with high social and economic costs; nearly every country faces a public health challenge, whether from undernutrition, overweight/obesity, and/or micronutrient deficiencies. Malnutrition is a multisector, multi-level problem that results from the complex interplay between household and individual decision-making, agri-food, health, and environmental systems that determine access to services and resources, and related policy processes. This paper reviewed the theory and recent qualitative evidence offered from 2010 to 2016 in the public health and nutrition literature on the role that agriculture plays in improving nutrition, how food systems are changing rapidly due to globalization, trade liberalization, and urbanization, and the implications this has for nutrition globally. The paper recommended leveraging Agri-food systems for nutrition by strengthening the institutional environment, making interventions more nutrition-sensitive, and developing the capacity to use improved decision-making [12]. Sobal Khan and Bisogni (1998) recommended a holistic perspective on the scope and scale of food and nutrition systems to strengthen social science work on agriculture, consumers, and health. Such a model integrates a conceptual model of the food and nutrition system, presents food and nutrition activities as part of a larger context, and identifies linkages among the many disciplines that deal with the food and nutrition system [13]. Mohajan and Haradhan (2014) recommended that implementing interventions by governments on food security, poverty reduction, transformation to commercial farming and agribusiness, markets, and the efficient use of agricultural products is the key to reducing food insecurity and malnutrition [14].

Masters et al., (2016) indicated that the nutrition transition in diets and health is closely related to other aspects of economic development, including agricultural transformation and urbanization as well as demographic change and epidemiological transitions from infectious to non-communicable diseases. Over time, dietary patterns in many countries have shifted from widespread inadequacy of many foods and nutrients, especially for children and mothers, to surplus energy intake and rising obesity with continued inadequacy of healthier foods. Diet-related diseases remain the single cause of premature death and disability in all regions. The authors combine food availability and dietary intake data from more than 100 countries over 30 years with a wide range of other evidence to characterize the nutrition transition and its association with changes in agricultural production and the food environment, asking how future dietary patterns might be steered towards healthier outcomes as national incomes grow. The paper concluded that more favorable prices for fruits and vegetables could facilitate increased intake, which benefits nutrition and health [15]. Maystadt Tan and Breisinger (2014) indicated the impact of price hikes and volatility on food security and concluded that only effective complementary policies against excessive global price volatility for net-food-importing countries could improve macro-level and household-level food security [16,17]. Popkin and Reardon (2018) studied food systems’ transformation and showed that Latin American Countries (LAC) are already among the global leaders in initiating demand-related solutions via taxation and marketing controls. Furthermore, shifting LAC’s food supply towards prices that incentivize the consumption of healthier diets and demand away from the less healthy component is not simple and will not happen immediately. The authors recommended that food industry firms must be incentivized to market the components of healthy diets. This can be implemented via selective taxes and subsidies, marketing controls, food quality regulations, and consumer education [18].

Previous studies’ surveys show the importance of agricultural food systems’ transformation to address malnutrition challenges in various regional settings across the world. Furthermore, the previous studies’ surveys were useful to formulate the regression model’s dependent and explanatory variables to be considered for both the undernourishment and obesity models adopted in this study.

The objective of this study is to specify the agricultural and food systems’ drives that contribute to malnutrition (undernourishment and obesity) in selected populous countries (India, Pakistan, and Egypt) and high-income countries in the Gulf Cooperation Council (GCC), Saudi Arabia, and the United Arab Emirates. The study aims to describe the policy actions needed to transform the agricultural and food sectors towards sustainable and healthy diets in the study’s countries addressing malnutrition challenges.

## 2. Materials and Methods

### 2.1. Data

We investigate the impact of specific indicators on the agricultural and food systems that contribute to malnutrition (undernourishment and obesity) in selected countries.

To build a wide food security information system, the Food and Agriculture Organization of the United Nations (FAO) had chosen indicators based on expert judgment and data availability with sufficient coverage to enable comparisons across regions and over time. Indicators are classified along the four dimensions of food security availability, access, utilization, and stability [4]. Definitions of the indicators/variable used in this study based on the FAO food security indicators suited are shown in Table 2. This study relied solely on published data from the Food and Agriculture Organization (FAO) and the World Bank sources and databases to build the econometrics model’s dependents and explanatory variables.

### 2.2. Method: Econometrics Models

Fixed- and random-effects models are usually employed when the number of cross-sectional units is large and the number of periods over which those units are observed is small [19].

Dummy variables are sometimes used in the context of panel or longitudinal data observations on a cross-section of individuals or firms over time. In this context, it is often assumed that the intercept varies across the N cross-sectional units and/or across the T periods. In the general case (N − 1) + (T − 1), dummies can be used for this, with computational shortcuts available to avoid having to run a regression with all these extra variables. This way of analyzing panel data is called the fixed-effects model. The dummy variable coefficients reflect ignorance—they are inserted merely for measuring shifts in the regression line arising from unknown variables [20].

Estimation with panel data allows us to control for individual heterogeneity, alleviate aggregation bias, improve efficiency by using data with more variability and less collinearity, estimate and test more complicated behavioral models, and examine adjustment dynamics.

Considering the panel regression model based on Gujarati [21] in Equation (1).

(1) $$Y_{𝒾𝓉}=\beta_{1𝒾}+\beta_{2}X_{2𝒾𝓉}+\beta_{3}X_{3𝒾𝓉}+\upsilon_{𝒾𝓉}$$

where Y stands for the dependent observed time-invariant heterogeneities, 𝒾 is the variable, 𝓉 is the time period, $\beta_{1𝒾}$ is the fixed effect, and υ is the error term.

Instead of treating $\beta_{1𝒾}$ as fixed, we assume that it is a random variable with a mean value of $\beta_{1}$ (no subscript 𝒾 here). The intercept value for an individual company can be expressed as in Equation (2).

(2) $$\beta_{1𝒾}=\beta_{1}+\varepsilon_{𝒾}\;𝒾=1,2,\ldots ,N$$

where $\varepsilon_{𝒾}$ is a random error term with a mean value of zero and variance of $\sigma .The_{\varepsilon }^{2}$ and the individual differences in the intercept values of each company are reflected in the error term $\varepsilon_{𝒾}$.

Substituting (2) into (1), we obtain Equations (3) and (4):

(3) $$Y_{𝒾𝓉}=\beta_{1}+\beta_{2}X_{2𝒾𝓉}+\beta_{3}X_{3𝒾𝓉}+\varepsilon_{𝒾}+\upsilon_{𝒾𝓉}\;=\beta_{1}+\beta_{2}X_{2𝒾𝓉}+\beta_{3}X_{3𝒾𝓉}+\omega_{𝒾𝓉}$$

where $\omega_{𝒾𝓉}=\varepsilon_{𝒾}+\upsilon_{𝒾𝓉}$

(4) $$\varepsilon_{𝒾}∼N(0,\sigma_{\varepsilon }^{2})\;\upsilon_{𝒾𝓉}∼N(\sigma_{\upsilon }^{2})\;E_{\left(\varepsilon_{𝒾}\upsilon_{𝒾𝓉}\right)}=0\;E_{\left(\varepsilon_{𝒾}\varepsilon_{j}\right)}=0\;𝒾\neq j\;E_{\left(\upsilon_{𝒾𝓉}\upsilon_{𝒾s}\right)}=E_{\left(\upsilon_{𝒾𝓉}\upsilon_{j𝓉}\right)}=E_{\left(\upsilon_{𝒾𝓉}\upsilon_{js}\right)}\;𝒾\neq j;\;𝓉\neq s$$

## 3. Results, Analysis, and Discussion

Descriptive statistics showed a wide variability of all dependent and explanatory variables used in this study with the highest variability in the variables total value of domestic food production variability and per capital value of domestic production, followed by the variability of food imports and per capita GDP—Table 3.

### 3.1. Prevalence of Undernourishment

In this study’s six selected countries’ prevalence of undernourishment, data from FAO showed that the most significant number of people is seen in India, exceeding 206 million people representing 15% of the country’s population, followed by Pakistan as the number of undernourished people reached 28 million people representing 13% percent of the country’s population. In 2020, other selected countries in this study witnessed undernourishment that varied from 8% in Oman, 5% in Egypt, and 4% of the population in both Saudi Arabia and UAE. Several countries report reductions in undernourishment. However, such progress has been reported to be slow and is not catching up with population growth—Table 4.

**Figure 4:**
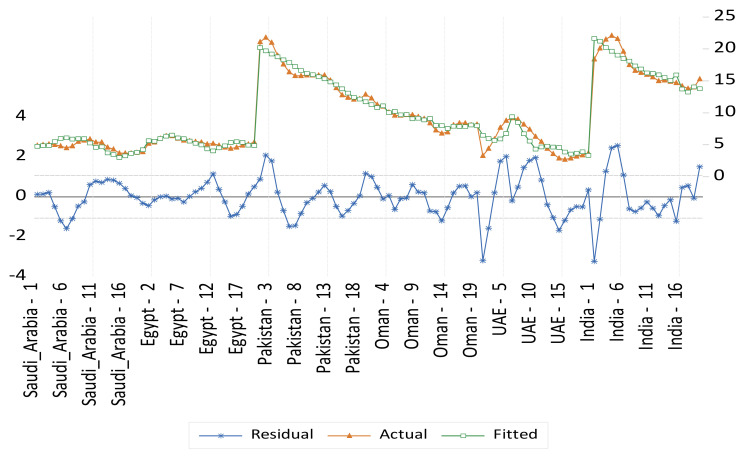
Actual, fitted, and residual of the undernourishment fixed-effects model.

**Figure 5:**
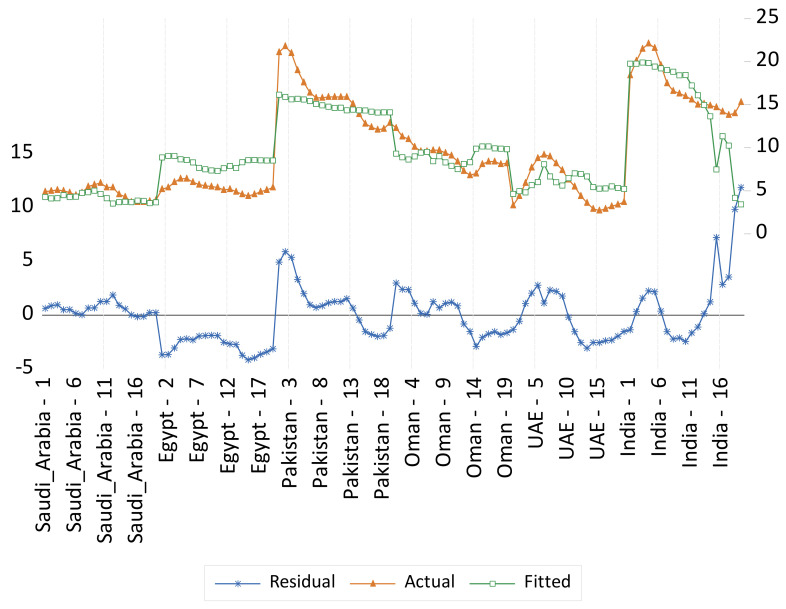
Actual, fitted, and residual of the undernourishment random-effects model.

Panel fixed- and random-effect models for undernourishment results are presented in Table 5 and illustrated in Figure 4 and Figure 5 respectively. Five variables were found to be significant in the fixed-effect model, and then these variables were considered for the random effect model. These variables are the food domestic production variability, per capita GDP, supply of fat, food losses at retail, and dummy variable representing a structural shift of the countries that witness a high level of urbanization relative to the other countries. Namely, Saudi Arabia, Oman, and UAE. These results indicate that food system variables such as food supply availability and food quality, the food supply chain development factors, and the level of urbanization are more significant determinants of undernourishment in the selected countries in this study. An agricultural sector transformation should not underestimate the importance of non-food production and consumption (food system) indirect factors (i.e., considering the food utilization dimension of food security). Food utilization is commonly understood as the way the body makes the most of various nutrients in the food. This food security dimension is determined primarily by people’s health status [22]. It is also important to consider non-food relevant factors such as income, the level of urbanization, people’s residence conditions, transportation to access fresh nutritious food, clean water and sanitation, energy resource availability, and the cost of transportation that enable people to access such resources. In agreement with previous studies, indirect food intake factors are highly important drivers to be considered for the transformation of the food sector to address malnutrition challenges. For example, as domestic production variability is reduced by 1%, it is expected that undernourishment would decrease by 0.07%. Meanwhile, a decrease in the supply of fat by 1% would reduce undernourishment by 0.11%. However, an increase in food losses at the retail level by 1% would increase undernourishment by 1.3%. There is a positive relationship between the rapid urbanization in Saudi Arabia, Oman, and UAE (selected GCC countries in this study) and the increase in malnutrition.

### 3.2. Prevalence of Obesity

**Figure 6:**
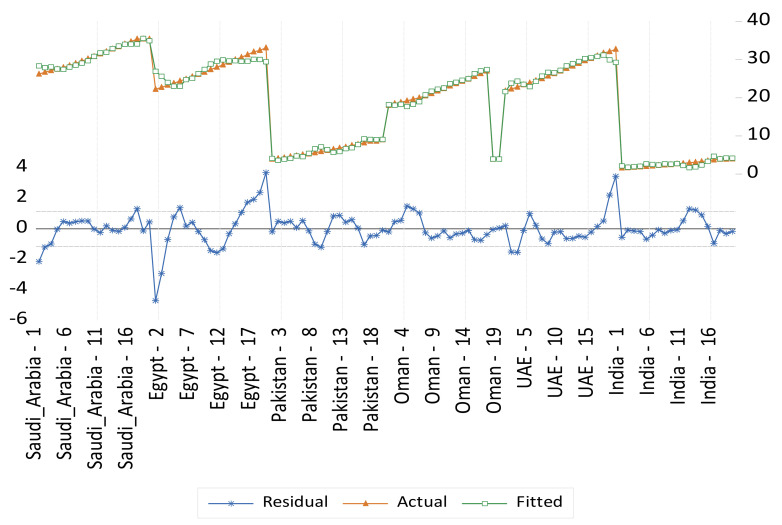
Actual, fitted, and residual of obesity in fixed-effect model.

**Figure 7:**
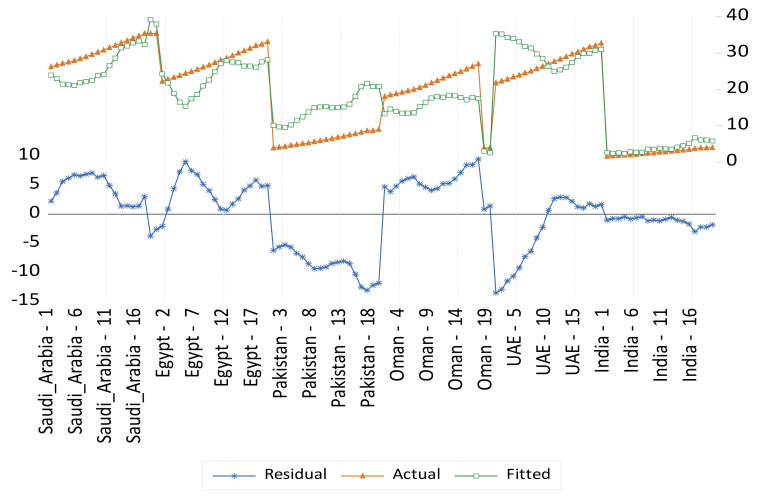
Actual, fitted, and residual of obesity in random-effect model.

The prevalence of obesity data from FAO showed that the prevalence of obesity is the highest in the United Arab Emirates as the prevalence reached 25%, followed by Saudi Arabia as the percentage reached 23% in 2020. The prevalence of obesity is relatively high in Egypt as it was reported to be 18% of the population in 2020. Meanwhile, obesity in Oman and Egypt was reported to be 18% in both countries. Pakistan was reported to have a 10% obesity rate in 2020—Table 6. Panel fixed- and random-effect models for obesity results are presented in Table 7 and illustrated in Figure 6 and Figure 7 respectively.

Panel fixed- and random-effect models for obesity results are presented in Table 7. Six variables were significant in the fixed-effect model and were then considered for the random-effect model. These variables are the dietary energy supply, per capita GDP, cereal import dependency, percentage of food import to export, supply of fat, and value of domestic production. For example, an increase in the cereal import dependency by 1% increased obesity by 0.15%. Meanwhile, an increase in the dietary energy supply by 1% increased obesity by 0.34%. Meanwhile, an increase in the value of domestic production (e.g., production of fresh local products as opposed to imported processed food) would reduce obesity by 0.01%. These results indicate that both nutrition and food system variables are highly significant in determining the overall obesity in this study’s selected countries.

Changes in food and nutrition systems around the world, including the six selected countries in this study, are causing several changes to the dynamics of dietary intake for consumers. Al Shamsi et al., 2018 showed that nutrition transition is a result of both demographic and socioeconomic factors [23]. First, consumers have shifted from traditional diets to westernized diets. Second, due to globalization, access to more food options including processed and ready-to-eat foods increased. Lastly, consumers changed their eating habits and adopted new food products. Authors noted a decline in cereal consumption and an increase in dependency on animal products such as butter, cheese, and animal protein rather than vegetables and legumes protein, all of which have been linked to the prevalence of non-communicable diseases such as type 2 diabetes and obesity consequences.

## 4. Conclusions

Malnutrition is a global challenge, including the burden of non-communicable diseases and large social and economic costs. Both undernourishment and obesity represent risk factors that hinder human well-being and national development. Gillespie and van den Bold (2017) showed that one in three people are affected, and every country in the world faces serious public health challenges due to malnutrition. The authors pointed out that the challenges constitute three dimensions, namely energy deficiency, micronutrient deficiency, and overweight or obesity [12].

Agricultural and food sectors’ transformation all over the world, including in the six countries selected in this study, aims to address malnutrition and non-communicable diseases that are imminent, including the need for a structural economic transformation to tackle poverty reduction through governmental support and budgetary provisions to rebalance agricultural sector subsidies, increase agricultural sector R&D, and promote of wide nutrient-rich food [7].

In this study, the results showed that the most influential factors of agricultural and food sector transformation, concerning undernourishment (highly significant using variables in the fixed- and random-effect models for the six selected countries), are the supply of fat from the nutrition side and the per capita GDP, urbanization, and flood losses throughout the supply chain from the agricultural and food systems side.

The most influential factors of agricultural and food sector transformation concerning obesity are the cereal import dependency, dietary energy supply, and supply of fat from the nutrition perspective. From the agricultural and food sector transformation perspective, variables found to be highly significant are the per capita GDP, percentage of food imports to exports, and the value of domestic production.

To ensure foods move along the value chains more efficiently, strategies must include improving accessibility, reducing costs, and lowering food losses in all six selected countries, which also apply to all other countries around the world. This can be achieved by defining the principles of engagement between the public and private sectors. Furthermore, there is a need to empower consumers to make more informed food choices, to satisfy the rising demand for sustainable healthy diets in the selected countries of India, Pakistan, Egypt, and the GCC countries included in this study (Saudi Arabia, Oman, and UAE).

In brief, food sector transformation includes support for food trade openness and tariff and non-tariff barrier reduction policies. Furthermore, such food and agriculture sectors’ transformation may support jobs across the food system including relevant services and marketing sectors, reduce food losses and waste, promote technology and financial innovation, and implement actions to assure safety nets and adjust taxes and subsidies on key food categories (e.g., plant-based food and reduced animals’ fat content food).

## Figures and Tables

**Table 1:** Matrix of food bilateral trade in million dollars in 2020 between the selected countries.

Exporting Country	Egypt	India	Oman	Pakistan	Saudi Arabia	UAE
Egypt	-	188	1	3	51	107
India	134		13	0.02	1	226
Oman	77	445		179	283	1015
Pakistan	71	38	1	-	7	443
Saudi Arabia	545	2607	132	327		6
UAE	240	1918	165	600	107	
Total	1067	5195	312	1109	449	1798

Source: FAO Stats [4].

**Table 2:** Definitions of the dependent and explanatory variables used in the study.

Variable	Definition
Prevalence of undernourishment (dependent variable 1) $Y_{1𝓉}$	The indicator estimates the proportion of the population whose habitual food consumption is insufficient to provide the dietary energy levels that are required to maintain a normally active and healthy life. It is calculated by applying the estimated prevalence of undernourishment to the total population in each period.
Prevalence of obesity in the adult population −18 years and older. (Dependent variable 2) $Y_{2𝓉}$	Percentage of adults aged 18 and over whose Body Mass Index (BMI) is more than 30 kg/m^2^. Obesity is also defined as an abnormally high percentage of body fat.
Gross domestic product per capita, PPP, dissemination (constant 2011 international $) $X_{1𝓉}$	GDP per capita based on purchasing power parity (PPP) is gross domestic product converted to international dollars using purchasing power parity rates. An international dollar has the same purchasing power over GDP as the U.S. dollar has in the United States. GDP at purchaser’s prices is the sum of gross value added by all resident producers in the economy plus any product taxes and minus any subsidies not included in the value of the products. It is calculated without making deductions for depreciation of fabricated assets or depletion and degradation of natural resources. Data are in constant 2017 international dollars. Per capita GDP (per capita income) is a variable of food *accessibility* variable and would be directly relevant to malnutrition.
The average value of food production (constant 2004–2006 I$/cap) (3-year average) $X_{2𝓉}$	The indicator expresses the food net production value (in constant 2004-2006 international dollars), as estimated by FAO and published by FAOSTAT, in per capita terms. The average value of food production is a variable of food *availability* and would be directly relevant to malnutrition.
Value of food imports in total merchandise exports (percent) (3-year average) $X_{3𝓉}$	Value of food (excluding fish) imports over total merchandise exports. The variable is relevant to the food *stability* dimension of food security.
Average dietary energy supply (percent) (3-year average) $X_{3𝓉}$	The indicator expresses the Dietary Energy Supply (DES). Each country’s or region’s average supply of calories for food consumption is normalized by the average dietary energy requirement estimated for its population to provide an index of adequacy of the food supply in terms of calories. Average dietary energy supply is a variable of *availability* and would be directly relevant to malnutrition.
The average supply of protein of animal origin (g/cap/day) (3-year average) $X_{4𝓉}$	National average protein supply (expressed in grams per caput per day). It includes the following groups: meat; offal; animal fats and products, milk, and products; eggs, fish, seafood, and products; and aquatic products, among others. Average supply of protein from animal sources is a variable of *availability* and would be directly relevant to malnutrition.
Average Fat Supply $X_{5𝓉}$	National average fat supply (expressed in grams per caput per day). The variable is *availability* and would be directly relevant to malnutrition.
Cereal import dependency ratio (percent) (3-year average) $X_{6𝓉}$	The indicator tells how much of the available domestic food supply of cereals has been imported and how much comes from the country’s production. It is computed as (cereal imports-cereal exports)/(cereal production + cereal imports-cereal exports) × 100. Given this formula, the indicator assumes only values ≤100. Negative values indicate that the country is a net exporter of cereals. The variable is food *accessibility* variable and would be directly relevant to malnutrition.
Per capita food production variability (constant 2004–2006 thousand int. $ per capita) $X_{7𝓉}$	The variability of the “food net per capita production value in constant 2004–2006 international $” as disseminated in FAOSTAT. The variable is food *stability* variable and would be directly relevant to malnutrition.
Incidence of caloric losses at the retail distribution level (percent) $X_{8𝓉}$	Incidence of caloric losses at the retail distribution level. The variable is *availability* and would be directly relevant to malnutrition.
Urbanization (Percentage of Total Population) $X_{9𝓉}$	Urban population refers to people living in urban areas as defined by national statistical offices. The data are collected and smoothed by United Nations Population Division. Several studies showed the importance of urbanization to assess food security, food, and agriculture transformation

**Table 3:** Descriptive statistics of the independent and dependent variables.

Variable	Unit	Mean	Std. Deviation	Coefficient of Variation (Std. Deviation/ Mean) %	Minimum	Maximum	Range
Prevalence of Undernourishment	%	9.6	5.5	57%	2.7	22.1	19.4
Prevalence of Obesity	%	19.1	11.5	60%	1.6	35.4	33.8
Per Capita GDP	Dollar/person	28,359	26,954	95%	2579	102,495	99,916
Value of Domestic Production	Dollar/person	195	327	168%	22	2616	2594
Percentage of Food Import/Export	%	13.2	12.8	97%	3.0	48.0	45.0
Dietary Energy Supply	Kcal/capita/day	51.4	10.0	19%	37.0	82.0	45.0
Supply of Animals Protein	gram/capita/day	38.0	23.7	62%	9.0	91.0	82.0
Supply of Fat	gram/capita/day	74.1	16.6	22%	44.7	108.4	63.7
Cereal Import Dependency	%	49.4	46.4	94%	−19.0	100.0	119.0
Food Production Variability	Thousand Dollar	10.1	14.7	145%	0.9	77.1	76.2
Food Losses at Retail	%	3.7	0.7	19%	2.4	5.4	2.9
Urbanization	% Total population	3.6	2.8	78%	0.5	15.6	15.1

**Table 4:** Mean value of the number of undernourished people per million for the period 2000 to 2020.

Country Name	Million People (2020)	Undernourished as a Percentage of the Total Population in the Country 2020	Mean in Million People (2000–2020)	Std. (Standard Deviation)	Coefficient of Variation (Std//Mean) %
Saudi Arabia	1.3	4%	1.3	0.17	13%
Egypt	5.4	5%	4.5	0.39	9%
Pakistan	27.9	13%	28.2	2.18	8%
Oman	0.4	8%	0.3	0.06	20%
UAE	0.4	4%	0.4	0.14	35%
India	208.6	15%	206.3	20.59	10%
Total	244	14%	241		

**Table 5:** Parameter estimates of undernourishment in fixed- and random-effect models results.

Variables	Parameter (β)		T-Stat		Sig. (*p*-Value)		Partial Eta Squared
	Fixed Effect Model	Random Effect Model	Fixed Effect Model	Random Effect Model	Fixed Effect Model	Random Effect Model	
Intercept	50.953	37.135	7.741	29.074	0.000	0.000	0.451
***Nutrition Variables:***							
Cereal Import Dependency	−0.016	-	−0.380	-	0.705	-	0.242
Cereal Import Dependency Shift	0.059	-	1.167	-	0.246	-	
DietaryEnergySupply	−0.112	-	−1.195	-	0.235	-	0.020
Supply of Animals Protein	−0.069	-	−1.310	-	0.193	-	0.016
Supply of Fat *	**−0.110**	**−0.096**	**−3.875**	**−6.082**	**0.000**	**0.000**	**0.264**
***Agricultural and Food Systems Transformation Variables:***							
Food Losses at Retail *	**1.278**	**−5.325**	**2.705**	**−22.847**	**0.008**	**0.000**	**0.001**
Food Production Variability *	−0.067	−0.009	−4.151	−0.500	0.000	0.618	**0.002**
Per Capita GDP *	**0.00012**	**0.00005**	**3.875**	**2.779**	**0.000**	**0.006**	**0.020**
Percentage of Food Import\Export	−0.058	-	−1.636	-	0.105	-	0.110
Urbanization	−1.681		−6.748		0.000	-	0.008
Urbanization Shift *	**1.603**	**−0.051**	**5.961**	**−5.398**	**0.000**	**0.000**	
Value of Domestic Production	0.001	-	1.299	-	0.197	-	0.006
[Country Code = 1]	−9.619	-	−3.459	-	<0.001	-	0.110
[Country Code = 2]	−2.403	-	−1.348	-	0.181	-	0.018
[Country Code = 3]	5.325	-	5.882	-	<0.001	-	0.263
[Country Code = 4]	−14.413	-	−6.663	-	<0.001	-	0.314
[Country Code = 5]	−11.451	-	−4.426	-	<0.001	-	0.168
[Country Code = 6]	0.000		.		.	.	.

* = Significant variables in the fixed-effect model. Bold and * = Significant variables in both fixed- and random-effect models. Fixed-effects model fitness. R Squared = 0.967980 (Adjusted R Squared = 0.962309). Random-effects model fitness. R Squared = 0.767602 (Adjusted R Squared = 0.756842). - indicate no paramater.

**Table 6:** Mean value of the number and percentages of obese people in million from 2000 to 2020.

Country Name	Million People (2020)	Obesity as a Percentage of the Total Population in the Country in 2020	Mean in Million People (2000–2020)	Std. (Standard Deviation)	Coefficient of Variation (Std//Mean) %
Saudi Arabia	8.1	23%	5.5	1.799	33%
Egypt	18.4	18%	12.9	3.066	24%
Pakistan	10.2	10%	6.3	2.557	41%
Oman	0.9	18%	0.7	0.827	118%
UAE	2.5	25%	1.6	0.756	47%
India	34.3	2%	21.6	8.478	39%
Total	74.40	4%	43.1		

**Table 7:** Parameter estimates of obesity using fixed- and random-effect models results.

Variables	Parameters (β)		T-Stat		Sig. (*p*-Value)		Partial Eta Squared
	Fixed Effect Model	Random Effect Model	Fixed Effect Model	Random Effect Model	Fixed Effect Model	Random Effect Model	
Intercept	−40.954	-	−5.779	-	0.000	-	0.372
***Nutrition Variables:***							
**Cereal Import Dependency**	**0.146**	**-**	**3.170**	**-**	**0.002**	**-**	**0.075**
Cereal Import Dependency Shift	−0.066	-	−1.213	-	0.228	-	
Dietary_Energy_Supply *	**0.344**	**0.209**	**3.415**	**10.849**	**0.001**	**0.000**	**0.186**
Supply of Animals Protein *	0.184	-	3.256	-	0.002	-	0.173
Supply of Fat *	**0.108**	**0.336**	**3.541**	**25.280**	**0.001**	**0.000**	**0.169**
***Agricultural And Food Systems Transformation Variables:***							
Food Losses at Retail	0.397	-	0.781	-	0.437	-	0.018
Food Production Variability	0.004	-	0.216	-	0.830	-	0.007
Per Capita GDP *	**0.173**	**0.456**	**4.540**	**32.478**	**0.000**	**0.000**	**0.081**
Percentage of Food Import\Export *	**−0.00013**	**0.00023**	**−3.766**	**28.024**	**0.000**	**0.000**	**0.150**
Urbanization	0.456	-	1.701	-	0.092	-	0.003
Urbanization Shift	−0.037	-	−0.127	-	0.899	-	-
Value of Domestic Production *	**−0.012**	**−0.007**	**−15.791**	**−14.793**	**0.000**	**0.000**	**0.686**
[Country Code = 1]	0.947	-	0.284	-	0.777	-	0.001
[Country Code = 2]	4.815	-	2.252	-	0.027	-	0.050
[Country Code = 3]	0.240	-	0.221	-	0.825	-	0.001
[Country Code = 4]	13.205	-	5.090	-	<0.001	-	0.211
[Country Code = 5]	16.761	-	5.402	-	<0.001	-	0.231
[Country Code = 6]	0.000	-	.	-	.	-	.

* = Significant Variable at the fixed effect. Bold and * = Significant Variable in both the fixed-effect and random-effect models. Fixed-Effect Model Fitness. R Squared = 0.991522 (Adjusted R Squared = 0.990020). Random-Effect Model Fitness. R Squared = 0.734165 (Adjusted R Squared = 0.721858).

## Data Availability

No supplemental data added.
